# Supplementary material for: Transcriptome Analysis to Explore the Cause of the Formation of Different Inflorescences in Tomato
Source: Int J Mol Sci. 2022 Jul 26;23(15):8216. doi: 10.3390/ijms23158216 (PMC9368726; doi:10.3390/ijms23158216)
Supplement: Supplementary file 1 [file ijms-23-08216-s001.zip › ijms-1789867-supplementary.pdf]

**Table S1A**. Functional classification of DEGs in E_SR-vs-E_CI

| GO_CFP Term Level2 | GO_CFP Term Level1 | num |
|---|---|---|
| metabolic process | biological_process | 117 |
| cellular process | biological_process | 106 |
| response to stimulus | biological_process | 48 |
| biological regulation | biological_process | 41 |
| regulation of biological process | biological_process | 37 |
| localization | biological_process | 16 |
| signaling | biological_process | 15 |
| cellular component organization or biogenesis | biological_process | 12 |
| developmental process | biological_process | 10 |
| multicellular organismal process | biological_process | 10 |
| reproduction | biological_process | 10 |
| reproductive process | biological_process | 10 |
| multi-organism process | biological_process | 4 |
| positive regulation of biological process | biological_process | 4 |
| negative regulation of biological process | biological_process | 3 |
| detoxification | biological_process | 3 |
| immune system process | biological_process | 1 |
| carbon utilization | biological_process | 1 |
| cell proliferation | biological_process | 1 |
| membrane | cellular_component | 97 |
| cell | cellular_component | 96 |
| cell part | cellular_component | 94 |
| membrane part | cellular_component | 91 |
| organelle | cellular_component | 66 |
| extracellular region | cellular_component | 16 |
| organelle part | cellular_component | 14 |
| protein-containing complex | cellular_component | 14 |
| cell junction | cellular_component | 2 |
| symplast | cellular_component | 2 |

**Table S1B**. Functional classification of DEGs in M_SR-vs-M_CI

| GO_CFP Term Level2 | GO_CFP Term Level1 | num |
|---|---|---|
| metabolic process | biological_process | 50 |
| cellular process | biological_process | 50 |
| response to stimulus | biological_process | 23 |
| biological regulation | biological_process | 12 |
| regulation of biological process | biological_process | 11 |
| localization | biological_process | 8 |
| signaling | biological_process | 7 |
| multicellular organismal process | biological_process | 5 |
| developmental process | biological_process | 5 |
| reproduction | biological_process | 5 |
| reproductive process | biological_process | 5 |
| multi-organism process | biological_process | 5 |
| cellular component organization or biogenesis | biological_process | 5 |
| negative regulation of biological process | biological_process | 2 |
| cell proliferation | biological_process | 1 |
| detoxification | biological_process | 1 |
| positive regulation of biological process | biological_process | 1 |
| membrane | cellular_component | 48 |
| cell | cellular_component | 46 |
| cell part | cellular_component | 46 |
| membrane part | cellular_component | 46 |
| organelle | cellular_component | 30 |
| protein-containing complex | cellular_component | 13 |
| extracellular region | cellular_component | 9 |
| organelle part | cellular_component | 8 |
| extracellular region part | cellular_component | 3 |
| membrane-enclosed lumen | cellular_component | 3 |
| binding | molecular_function | 85 |
| catalytic activity | molecular_function | 71 |

**Table S1C**. Functional classification of DEGs in L_SR-vs-L_CI

| GO_CFP Term Level2 | GO_CFP Term Level1 | num |
|---|---|---|
| metabolic process | biological_process | 399 |
| cellular process | biological_process | 388 |
| biological regulation | biological_process | 170 |
| regulation of biological process | biological_process | 148 |
| response to stimulus | biological_process | 116 |
| localization | biological_process | 63 |
| cellular component organization or biogenesis | biological_process | 58 |
| signaling | biological_process | 42 |
| developmental process | biological_process | 35 |
| multicellular organismal process | biological_process | 29 |
| positive regulation of biological process | biological_process | 23 |
| reproduction | biological_process | 20 |
| reproductive process | biological_process | 20 |
| negative regulation of biological process | biological_process | 19 |
| multi-organism process | biological_process | 17 |
| detoxification | biological_process | 7 |
| immune system process | biological_process | 4 |
| cell killing | biological_process | 2 |
| growth | biological_process | 2 |
| carbon utilization | biological_process | 1 |
| cell proliferation | biological_process | 1 |
| pigmentation | biological_process | 1 |
| rhythmic process | biological_process | 1 |
| membrane | cellular_component | 389 |
| membrane part | cellular_component | 371 |
| cell | cellular_component | 325 |
| cell part | cellular_component | 313 |
| organelle | cellular_component | 239 |
| protein-containing complex | cellular_component | 55 |

**Table S2A**. KEGG channel data of DEGs were analyzed in E_SR-vs-E_CI comparison.

| KEGG Pathway Term Level2 | KEGG Pathway Term Level1 | num |
|---|---|---|
| Transport and catabolism | Cellular Processes | 12 |
| Cell growth and death | Cellular Processes | 2 |
| Signal transduction | Environmental Information Processing | 13 |
| Membrane transport | Environmental Information Processing | 2 |
| Translation | Genetic Information Processing | 14 |
| Folding, sorting and degradation | Genetic Information Processing | 11 |
| Replication and repair | Genetic Information Processing | 2 |
| Transcription | Genetic Information Processing | 1 |
| Biosynthesis of other secondary metabolites | Metabolism | 31 |
| Global and overview maps | Metabolism | 24 |
| Carbohydrate metabolism | Metabolism | 23 |
| Amino acid metabolism | Metabolism | 20 |
| Metabolism of terpenoids and polyketides | Metabolism | 16 |
| Energy metabolism | Metabolism | 13 |
| Metabolism of other amino acids | Metabolism | 13 |
| Lipid metabolism | Metabolism | 12 |
| Metabolism of cofactors and vitamins | Metabolism | 7 |
| Nucleotide metabolism | Metabolism | 5 |
| Glycan biosynthesis and metabolism | Metabolism | 4 |
| Environmental adaptation | Organismal Systems | 6 |
| Aging | Organismal Systems | 1 |

**Table S2B**. KEGG channel data of DEGs were analyzed in M_SR-vs-M_CI comparison.

| KEGG Pathway Term Level2 | KEGG Pathway Term Level1 | num |
|---|---|---|
| Transport and catabolism | Cellular Processes | 11 |
| Cell growth and death | Cellular Processes | 6 |
| Signal transduction | Environmental Information Processing | 8 |
| Membrane transport | Environmental Information Processing | 2 |
| Translation | Genetic Information Processing | 12 |
| Folding, sorting and degradation | Genetic Information Processing | 11 |
| Replication and repair | Genetic Information Processing | 3 |
| Transcription | Genetic Information Processing | 3 |
| Carbohydrate metabolism | Metabolism | 18 |
| Biosynthesis of other secondary metabolites | Metabolism | 11 |
| Global and overview maps | Metabolism | 9 |
| Energy metabolism | Metabolism | 9 |
| Amino acid metabolism | Metabolism | 8 |
| Lipid metabolism | Metabolism | 7 |
| Metabolism of cofactors and vitamins | Metabolism | 4 |
| Metabolism of other amino acids | Metabolism | 4 |
| Metabolism of terpenoids and polyketides | Metabolism | 3 |
| Nucleotide metabolism | Metabolism | 2 |
| Environmental adaptation | Organismal Systems | 9 |
| Aging | Organismal Systems | 2 |

**Table S2C**. KEGG channel data of DEGs were analyzed in L_SR-vs-L_CI comparison.

| KEGG Pathway Term Level2 | KEGG Pathway Term Level1 | num |
|---|---|---|
| Transport and catabolism | Cellular Processes | 42 |
| Cell growth and death | Cellular Processes | 13 |
| Signal transduction | Environmental Information Processing | 62 |
| Membrane transport | Environmental Information Processing | 6 |
| Folding, sorting and degradation | Genetic Information Processing | 43 |
| Translation | Genetic Information Processing | 43 |
| Transcription | Genetic Information Processing | 16 |
| Replication and repair | Genetic Information Processing | 8 |
| Carbohydrate metabolism | Metabolism | 85 |
| Global and overview maps | Metabolism | 69 |
| Lipid metabolism | Metabolism | 67 |
| Amino acid metabolism | Metabolism | 52 |
| Biosynthesis of other secondary metabolites | Metabolism | 47 |
| Energy metabolism | Metabolism | 39 |
| Metabolism of terpenoids and polyketides | Metabolism | 36 |
| Metabolism of other amino acids | Metabolism | 30 |
| Metabolism of cofactors and vitamins | Metabolism | 27 |
| Nucleotide metabolism | Metabolism | 15 |
| Glycan biosynthesis and metabolism | Metabolism | 6 |
| Environmental adaptation | Organismal Systems | 37 |
| Aging | Organismal Systems | 2 |

**Table S3**. 14 pairs of primers for qRT-PCR

| Primer | 5'to3' |
|---|---|
| S-CHS2 | GTGCAAAGGGACCAGCTACT |
| | TCACCTGCGTTAAACGAAAAAT |
| S-ARF4 | GTGAGCAAATGTCGCCTTGG |
| | GTTTTCCCTGCTTTCATGGTGT |
| S-AN | TGCAAGATTCAAGCCCCGAT |
| | GCAATGCCCAAAGCCTCAAA |
| S-FA | AAGCGAGAGACAAAGGGAGC |
| | ACGAGGTCCTCTGTTACCAC |
| S-SP | TGTGAACCCCTTGTGATTGGT |
| | TGATCATGTCATTTCCCCTTCCA |
| S-blind | GGGAAGAGCTCCATGTTGTGA |
| | AATTGCATTTCTTTCTGAACCCT |
| S-LOC101243791 | AATTGCATTTCTTTCTGAACCCT |
| | AGCATGAGCGACTGCAAAGA |
| S-Cwp | GGAGTTTGCAAAAGGGCTGG |
| | GGAGTTTGCAAAAGGGCTGG |
| S-F3H | GGAGTTTGCAAAAGGGCTGG |
| | TGGCTCCACCATTATTTCGTACT |
| S-krp2 | TGGCTCCACCATTATTTCGTACT |
| | GCGGGGTCTCGTATTGAGAG |
